# Confronting the known unknown: historical lessons and future strategies for Disease X

**DOI:** 10.3389/fpubh.2026.1833807

**Published:** 2026-06-05

**Authors:** Yunhui Xiang, Guokang Sun, Pinpin Xiang, Jiangtao Hu, Lvbo Tian, Qin Zhang, Junxian Wang, Chunbao Xie

**Affiliations:** 1 Department of Laboratory Medicine and Port Epidemic Disease Monitoring Key Laboratory of Sichuan Province, Sichuan International Travel Healthcare Center (Port Clinic of Chengdu Customs), Chengdu, China; 2 Department of Laboratory Medicine, West China School of Public Health and West China Fourth Hospital of Sichuan University, Chengdu, China; 3 Department of Laboratory Medicine, Xiping Community Healthcare Center of Longquanyi District, Chengdu, China; 4 Department of Laboratory Medicine and Genetic Diseases Key Laboratory of Sichuan Province, Sichuan Provincial People's Hospital and University of Electronic Science and Technology of China, Chengdu, China

**Keywords:** Disease X, equity, non-pharmaceutical intervention, pandemic, therapy, vaccine, virology

## Abstract

The 2026 outbreak of Nipah virus in West Bengal, India, a known WHO priority pathogen, serves as a timely reminder of the constant threat from high-consequence emerging diseases and underscores the critical relevance of Disease X—a future pandemic caused by an unknown pathogen. The profound global health and economic impact of COVID-19 has made the potential devastation of Disease X tangible. However, despite the insights gained from COVID-19, significant gaps remain in translating this awareness into an effective, coordinated, and pre-emptive preparedness strategy. This review advances an integrated response framework built around a One Health surveillance diagnostic therapeutic continuum. This foundational system enables real-time pathogen tracking and host immune profiling across human, animal, and environmental interfaces. Insights generated through this continuum directly inform the timely activation of non-pharmaceutical interventions, guide dynamic modeling, and enable the precise deployment of rapid medical countermeasures. Furthermore, these measures should be supported by resilient health infrastructure and equitable data sharing mechanisms, and be embedded within a governance model that prioritizes global collaboration and equity. Ultimately, mitigating Disease X requires a unified strategy that seamlessly integrates continuous monitoring, diagnostics, and treatment within a resilient and equitable operational architecture, thereby transforming pandemic preparedness from concept into reality.

## 1 Introduction

The persistent threat of high-consequence pathogens and the potential for novel agents to trigger global health crises define the contemporary pandemic landscape. This reality is underscored by the recurrent emergence of known priority diseases, as exemplified by a 2026 Nipah virus outbreak in India, and by the historical impact of novel pathogens like SARS-CoV-2. The COVID-19 pandemic served as a concrete manifestation of the Disease X concept—a formal framework established by the World Health Organization (WHO) to represent a serious epidemic caused by a pathogen currently unknown to cause human

disease (1, 2). The inclusion of this placeholder on the WHO priority list alongside known threats encapsulates the core challenge of modern preparedness: building robust defenses against identified pathogens while developing systemic resilience against the unknown (1).

The urgency of such a framework stems from the profound, multi-systemic burden a Disease X-level event would impose—an impact extending far beyond acute mortality to ignite a compound global crisis. The direct health impacts are undeniably severe, characterized not only by substantial mortality—as evidenced by the WHO recording ~779.2 million COVID-19 cases and ~7.1 million deaths by April 2026—but also by acute morbidity and complex long-term sequelae, such as Ebola-related neurological complications and Long COVID (3, 4). Furthermore, novel pathogens intricately interact with pre-existing conditions, complicating clinical management through mechanisms like host immune dysregulation (5). These health shocks rapidly cascade into catastrophic economic and social repercussions. The COVID-19 pandemic, for instance, triggered the most severe global recession since 1929, with global GDP contracting by 3% and international trade declining by 7% in 2020 (6, 7). This economic turmoil disrupted global food systems, pushing an additional 83–132 million people into undernourishment in 2020, alongside the 690 million already food-insecure (8). Protectionist measures and logistical breakdowns drove food price spikes that devastated low-income countries, exacerbating social instability and health inequities in a vicious cycle (9). Concurrently, the mental health and educational tolls have been immense. The pandemic drove a significant global rise in depressive and anxiety disorders, particularly among vulnerable demographics like adolescents (10–12). Educational disruptions were equally staggering: at the peak of nationwide closures in April 2020, UNESCO estimated that 1.6 billion students across 200 countries-−94% of the global student population—were out of school. The subsequent failure of roughly one-third of countries to implement learning recovery measures post-closure only deepened educational inequalities along socioeconomic lines (13). Compounding these societal fractures, the pandemic placed unprecedented strain on global governance and multilateral cooperation. At a time when coordinated action was paramount, political polarization, declining institutional trust, and the proliferation of misinformation hampered public health responses. Furthermore, the prioritization of unilateral domestic measures over international coordination, alongside the politicization of the crisis, constrained the efficacy of multilateral institutions like the WHO and fragmented global solidarity. Beyond the socioeconomic and political spheres, the crisis yielded unintended environmental consequences that underscore the interconnectedness of ecological and human health (14). Although lockdowns temporarily improved air quality in certain regions, they simultaneously disrupted recycling infrastructures and spurred a reliance on single-use plastics, highlighting complex trade-offs between immediate public health mandates and long-term environmental sustainability (15). Underlying all these dimensions is the pandemic's fundamental nature as an “inequality virus”. The burden of crisis is never evenly distributed: marginalized ethnic groups suffered disproportionately higher infection and mortality rates driven by systemic racism and socioeconomic disadvantage, while global wealth disparities widened starkly (10, 16). This intertwined devastation demonstrates that a pandemic acts not as an equalizer, but as a potent amplifier of pre-existing inequities rooted in race, class, and resource access. Crucially, these cascading crises are deeply coupled; each shock feeds into the next, demanding a holistic, integrated preparedness strategy (17).

In response to these interconnected challenges, this review synthesizes contemporary advances to offer a comprehensive perspective on pandemic preparedness that extends beyond any single pathogen. Building on lessons from past outbreaks and the clear threats demonstrated by recent events, we systematically examine the potential origins of Disease X, evaluate key accelerating drivers, and propose a spectrum of preparedness strategies—ranging from surveillance and medical countermeasures to global governance. By emphasizing the integration of scientific evidence into coordinated public health action, this analysis bridges foundational research with practical implementation, distilling lessons from known threats into a proactive, pathogen-agnostic strategy designed to mitigate the ultimate unknown threat.

## 2 Possible origins and accelerating drivers

### 2.1 Natural spillover from zoonotic reservoirs

Both NiV and SARS-CoV-2 highlight the primary zoonotic spillover pathway, underscoring the persistent pandemic risk at the animal-human interface. High-risk interfaces are frequently driven by commercial wildlife trade and consumption, with systematic reviews documenting the prevalence of diverse zoonotic pathogens in wild meat animals and providing direct epidemiological links to human infection (18, 19). Viral evolution, including mutations that enhance host adaptation—such as those in the spike protein of coronaviruses for human cell receptor binding—further facilitates this crossover. High-pathogenicity avian influenza viruses exemplify this threat through unique mechanisms that can compromise human defenses (20–22). Transmission operates at complex interfaces, including settings like wildlife rehabilitation centers, where zoonotic transmission of pathogens like Chlamydia psittaci from birds to staff has been documented (23).

### 2.2 Environmental crime as a risk multiplier and pathway

A critical and structurally distinct risk multiplier lies in environmental crime, which creates volatile ecological interfaces often beyond the reach of regulated oversight and surveillance, representing a significant blind spot for “unknown unknowns”. Illegal activities such as deforestation, unregulated mining, and wildlife trafficking degrade ecosystems and establish high-contact zones in remote areas with fragile healthcare systems (24, 25). For instance, the COVID-19 pandemic exacerbated economic pressures, leading to increased illegal timber extraction and poaching in biodiverse nations, thereby elevating pathogen exposure risk (26). Rapid deforestation and unprecedented wildlife trafficking are pivotal in triggering zoonotic spillover by destroying biodiversity and forcing vulnerable human populations into close contact with wildlife reservoir hosts (25). The illegal wildlife trade itself is a vast reservoir; smuggled Sunda pangolins have shown serological evidence of exposure to SARS-related coronaviruses, illustrating the direct risk along trafficking pathways (27). Furthermore, illegal pet trade, such as that of parrots, can introduce and spread invasive pathogens, posing threats to native wildlife and potentially humans (28). These illicit activities often converge with informal settlements and illicit economies, creating complex socio-ecological risk landscapes with minimal surveillance. In such settings, community health and resilience are undermined by issues like a lack of safe sanitation, which increases disease risk and exposure to sanitation-related violence (29), and illicit drug trafficking, which elevates the risk of blood-borne disease outbreaks (30). A historical perspective reveals that concerns over species invasions as a major threat to global health have existed long before modern disease ecology frameworks (31).

Meanwhile, preventive ecological interventions fundamentally reduce spillover risks by altering the incentives for environmental crime and blocking pathogen transmission before it occurs. This shifts the paradigm from passive detection to the proactive elimination of ecological and socioeconomic drivers. For instance, stringent enforcement of the Convention on International Trade in Endangered Species of Wild Fauna and Flora curtails illegal wildlife trade, thereby lowering the risk of pathogen introduction into human populations (32). Similarly, habitat conservation and comprehensive land-use planning rebuild natural buffers. Restoring degraded wetlands reestablishes ecological separation between migratory birds and poultry, mitigating avian influenza risk, while retaining large forests and establishing buffer zones around settlements maintains spatial barriers among wildlife, livestock, and humans to slow deforestation-driven spillover (33, 34). For vector-borne diseases, environmental management—such as eliminating mosquito breeding sites and restoring natural drainage—effectively reduces community-level transmission (35). Finally, regulating wildlife markets through stricter hygiene standards, reduced animal crowding, and rigorous authorized inspections significantly lowers associated public health risks (36, 37).

### 2.3 Engineered pathogens: accidental or deliberate release

Modern biotechnology presents a distinct, non-zoonotic pathway for the emergence of Disease X through the intentional or accidental release of engineered pathogens—another domain where “unknown unknowns” may arise from rapid technological advances outpacing governance (38). The tangible threat of unintended escape is evidenced by persistent laboratory-acquired infections, even in high-income settings, highlighting risks to both personnel and the community (39, 40). A comprehensive threat assessment should recognize a natural-anthropogenic continuum. A compiled dataset of 71 high-risk, human-caused pathogen exposure events between 1975 and 2016 demonstrates that incidents resulting entirely from human decisions and mistakes are varied and significant (41). This continuum blurs the lines between natural spillover, accidental research leaks, and deliberate misuse, complicating detection and response.

### 2.4 Accelerating systemic drivers

The emergence of Disease X is propelled by a convergence of accelerating systemic drivers that amplify both the likelihood of a pathogen spillover and the potential severity of its impact. Fundamentally, this threat is rooted in zoonotic pathogens, whose risks are systemically heightened by climate change and anthropogenic environmental alterations. Climate change drives the redistribution of viral hosts and vectors, expands disease geographic ranges, and extends transmission windows, while simultaneously straining public health infrastructure through economic losses and—alongside conflicts and food crises—fueling mass displacement. These dynamics create ideal conditions for pathogens to exploit weakened systems and globalized networks (42, 43). Concurrently, habitat destruction and expanding urbanization intensify human-animal contact and create dense population nodes that accelerate transmission, with global mobility then enabling rapid cross-border spread of initially localized outbreaks (42, 44). Pathogen dissemination is further facilitated through environmental contamination, such as *via* agricultural runoff or the release of preserved microbes from thawing permafrost, illustrating transmission across interconnected human-animal-environment interfaces (45–47). Critical surveillance gaps, including an over-reliance on targeted diagnostics like PCR and ELISA that fail to detect novel pathogens, further elevate the oversight risk for potential Disease X candidates (19, 38).

Beyond these ecological pressures, the intrinsic adaptability of viruses constitutes a core accelerant. Establishing efficient human transmission requires overcoming molecular and cellular barriers, achieved through multifaceted adaptations. For influenza viruses, key mutations enable the viral polymerase to utilize mammalian host factors like ANP32 proteins, overcoming a major replication restriction (48). Viruses also subvert host machinery; for instance, influenza can exploit the host exonuclease TREX1 to blunt the innate immune response (49). Proteolytic activation of viral surface glycoproteins, such as through acquiring multibasic cleavage sites and adapting to host proteases like TMPRSS2, is another pivotal determinant of zoonotic potential and transmissibility for both influenza and coronaviruses (50). These adaptive pathways are influenced by viral subtype, receptor binding, and functional balance between proteins, offering multiple evolutionary routes to efficient spread (51). The complexity of host-pathogen interactions is exemplified by SARS-CoV-2, which employs a vast network of interactions with host factors for entry, replication, and immune evasion (52). Viral evolution, however, is constrained by lineage-specific genetic backgrounds. As seen with Chikungunya virus, key adaptive mutations in one lineage may not confer advantage—or can even be detrimental—in another, shaped by epistatic interactions, adding a layer of complexity in predicting emergence pathways (53).

Finally, the transmissibility of respiratory pathogens is modulated by ecological context. Respiratory viruses are expelled within a consortium of commensal bacteria, such as *Staphylococcus aureus* and *Streptococcus pneumoniae*, which can enhance viral persistence in droplets and aerosols through biophysical changes. This suggests an individual's respiratory microbiome is an underappreciated factor influencing the environmental stability and transmission efficiency of viruses (54).

## 3 Potential pathogen spectrum

Current evidence and expert assessments, informed by evolving frameworks such as the WHO's 2024 scientific blueprint for epidemic and pandemic research preparedness, emphasize that the potential sources of a Disease X event span a broad biological spectrum. This updated strategy moves beyond listing individual pathogens by advocating for a proactive, family-level approach to research and preparedness, thereby operationalizing the abstract notion of Disease X into a dynamic, scalable framework for risk identification and mitigation across the entire pathogen landscape (55). By prioritizing research on entire pathogen families rather than a few individual pathogens, this strategy enhances the ability to effectively respond to unforeseen variants, emerging pathogens, zoonotic transmission, and unknown threats. Furthermore, by designating prototype pathogens as models, research can generate generalizable evidence and fill knowledge gaps, thereby facilitating the development of medical countermeasures—including vaccines, therapeutics, and diagnostic tools—against other pathogens within the same family. While the WHO's prioritized pathogen list has historically focused on viral and bacterial agents, the distinct and frequently overlooked risks posed by fungal, prion, and parasitic pathogens necessitate their consideration in a comprehensive assessment of Disease X potential.

Respiratory viruses remain at the forefront of Public Health Emergency of International Concern (PHEIC) due to their efficient transmission and historical impact. Influenza A viruses, including subtypes such as H5N1, represent a persistent and high-risk pandemic threat given their continuous evolution (56, 57). Similarly, coronaviruses—encompassing pathogens like SARS-CoV-2 and MERS-CoV—have demonstrated a clear capacity to cause global outbreaks (58). Beyond respiratory viruses, several high-consequence viral families exhibit alarming Disease X potential. Filoviridae (e.g., Ebola and Marburg viruses) and Paramyxoviridae (e.g., Nipah virus) are classified as high risk due to their severity, zoonotic origin, and potential for severe societal disruption (55). Evolutionary studies within these families, such as those noting increased human-transmission potential in Marburg virus, highlight the need for pathogen-specific risk evaluation (59). Arboviruses also represent a rapidly expanding threat. Families such as Flaviviridae (e.g., dengue and Zika viruses) and Togaviridae (e.g., chikungunya virus) are considered high risk, characterized by adaptive mutations that enhance vector competence and facilitate global spread—a pattern intensified by climate change and globalization (60). Well-known pathogens like dengue and Zika viruses remain on the list despite established control measures, primarily due to their intrinsic transmissibility, virulence, and ongoing gaps in medical countermeasures. Dengue poses a heightened risk of severe disease via antibody-dependent enhancement (ADE), wherein secondary infection with a different serotype exacerbates illness; furthermore, current vaccines carry safety risks for seronegative individuals, and specific antivirals are lacking (61). Zika virus, which precipitated the 2015–2016 PHEIC and is linked to severe congenital abnormalities like microcephaly, persists as a threat. Despite post-outbreak herd immunity, viral circulation continues, with the re-accumulation of susceptible populations and the geographic expansion of mosquito vectors, Zika retains the potential to trigger future PHEICs (62).

Within the WHO assessment framework, bacteria are collectively rated as posing a “high” PHEIC risk, as

“highly pathogenic bacteria with high-level enteric or respiratory spread have caused and will cause severe outbreaks.”

Thus, bacterial pandemics remain a persistent threat. Priority pathogens include *Vibrio cholerae* serogroup O139, *Yersinia pestis, Shigella dysenteriae* serotype 1, *Salmonella enterica non-typhoidal serovars*, and *Klebsiella pneumoniae*. Cholera remains highly epidemic-prone in resource-limited settings; the emergence of O139—capable of replacing or co-circulating with O1—could shift epidemic patterns. Despite a bivalent whole-cell vaccine, coverage remains challenging, and control relies on rehydration and antibiotics, which rising resistance increasingly threatens (63). *Yersinia pestis*, the agent of plague, poses a persistent threat, primarily via pneumonic transmission; while early antibiotics are effective, rapid disease progression and the lack of a widely available, highly effective vaccine underscore its pandemic potential (64). *Shigella dysenteriae* serotype 1 remains a leading cause of pediatric diarrheal mortality; its fecal–oral transmission, growing fluoroquinolone resistance, and lack of a vaccine could precipitate severe outbreaks (65). *Salmonella enterica non-typhoidal serovars*, primarily foodborne, cause systemic infections in immunocompromised individuals, with the WHO identifying their rising antibiotic resistance as a critical concern despite ongoing vaccine development (66). Finally, *Klebsiella pneumoniae*, a leading carbapenem-resistant Enterobacterales, increasingly causes untreatable healthcare-associated infections. While novel antibiotics and vaccines are in development, limited access and the potential for highly resistant strains to achieve community transmission pose a serious emergency risk (67).

Historically, fungi have rarely sparked global pandemics compared to viruses and bacteria, primarily due to limited respiratory person-to-person transmission. Nevertheless, the WHO Fungal Priority Pathogens List (2022) highlights a rising burden of invasive fungal diseases, driven largely by expanding immunocompromised populations resulting from medical advances, HIV, and cancer therapies. While certain fungi possess outbreak potential, emerging antifungal resistance increasingly complicates treatment (68). *Candida auris* exemplifies this growing threat: this multidrug-resistant pathogen exhibits extensive resistance across antifungal classes, high mortality in invasive infections, and persistent nosocomial transmission due to environmental resilience (69). Its near-simultaneous emergence of distinct clades across continents underscores an adaptive versatility mirroring key Disease X attributes—novel origins, transmissibility potential, and profound diagnostic and therapeutic challenges (70). Consequently, the WHO designates *Candida auris* a critical-priority pathogen, urging enhanced surveillance and countermeasure development (68). The three other critical-priority fungi—*Cryptococcus neoformans, Aspergillus fumigatus*, and *Candida albicans*—lack efficient airborne or interhuman transmission, making acute respiratory-style pandemics unlikely. Instead, their primary threat stems from limited antifungal access and emerging resistance; against a backdrop of expanding immunocompromised populations, they are more likely to exert a sustained global health impact as an “endemic pandemic.”

Prions represent a unique class of infectious agents consisting solely of misfolded proteins that cause fatal neurodegenerative diseases (71). Although recent preclinical studies have shown promise, no disease-modifying therapies have yet demonstrated significant clinical efficacy in humans, and no vaccines are currently available (72). Prion transmission is highly restricted, typically requiring direct ingestion of infected tissue or iatrogenic exposure, with human-to-human transmission being exceedingly rare. However, prions can persist in the environment for years and are resistant to conventional disinfection. Cross-species transmission events, such as variant Creutzfeldt-Jakob disease, have been documented (73). Moreover, scavengers influence the geographical spread of prions through complex ecological interactions, which may either amplify or reduce transmission risks (74). Current surveillance may significantly underestimate cases, and humans remain underprepared in terms of diagnosis, treatment, and public awareness (73, 75). Furthermore, the potential weaponization of prions adds critical complexity to biosafety and biodefense frameworks (76).

Parasites, comprising unicellular protozoa and multicellular helminths, exhibit diverse transmission modes and epidemic potential. While no parasite has historically caused a global pandemic, certain species have spread regionally alongside human migration and retain the capacity for cross-border outbreaks (77). Among protozoa, *Plasmodium* is not listed as a WHO priority pathogen for pandemics yet retains significant outbreak potential. Although its reliance on Anopheles mosquitoes restricts transmission compared to respiratory viruses, climate change is expanding vector ranges and elevating risk. Furthermore, emerging artemisinin resistance, diagnostic gaps, and fragile supply chains could overwhelm response capacities during outbreaks (78). *Cryptosporidium* and *Giardia* cause waterborne outbreaks via the fecal–oral route, distinguished by high disinfection resistance and limited therapeutic options; documented treatment failures and the absence of vaccines further complicate control (79). *Microsporidia*, obligate intracellular parasites, cause severe disease in immunocompromised hosts and are designated Category B priority pathogens by the U.S. National Institute of Allergy and Infectious Diseases (80). *Toxoplasma gondii* typically persists as an asymptomatic latent infection with low virulence, precluding pandemic potential, though it poses specific risks to fetuses and immunocompromised populations (81). *Leishmania* species, transmitted by sandflies, remain largely confined to specific geographic regions due to ecological constraints, resulting in a relatively low outbreak risk (82, 83). Conversely, multicellular helminths—such as Schistosoma species (dependent on aquatic snail intermediate hosts), Taenia species (requiring livestock intermediate hosts), and vector-borne filarial nematodes—pose a substantially lower pandemic risk due to complex life cycles that hinder rapid human-to-human spread. Infections are typically chronic with low acute mortality, and effective, low-cost drugs facilitate large-scale preventive chemotherapy (84). Nevertheless, emerging drug resistance drives treatment failures and persistent transmission (85).

**Figure 1:**
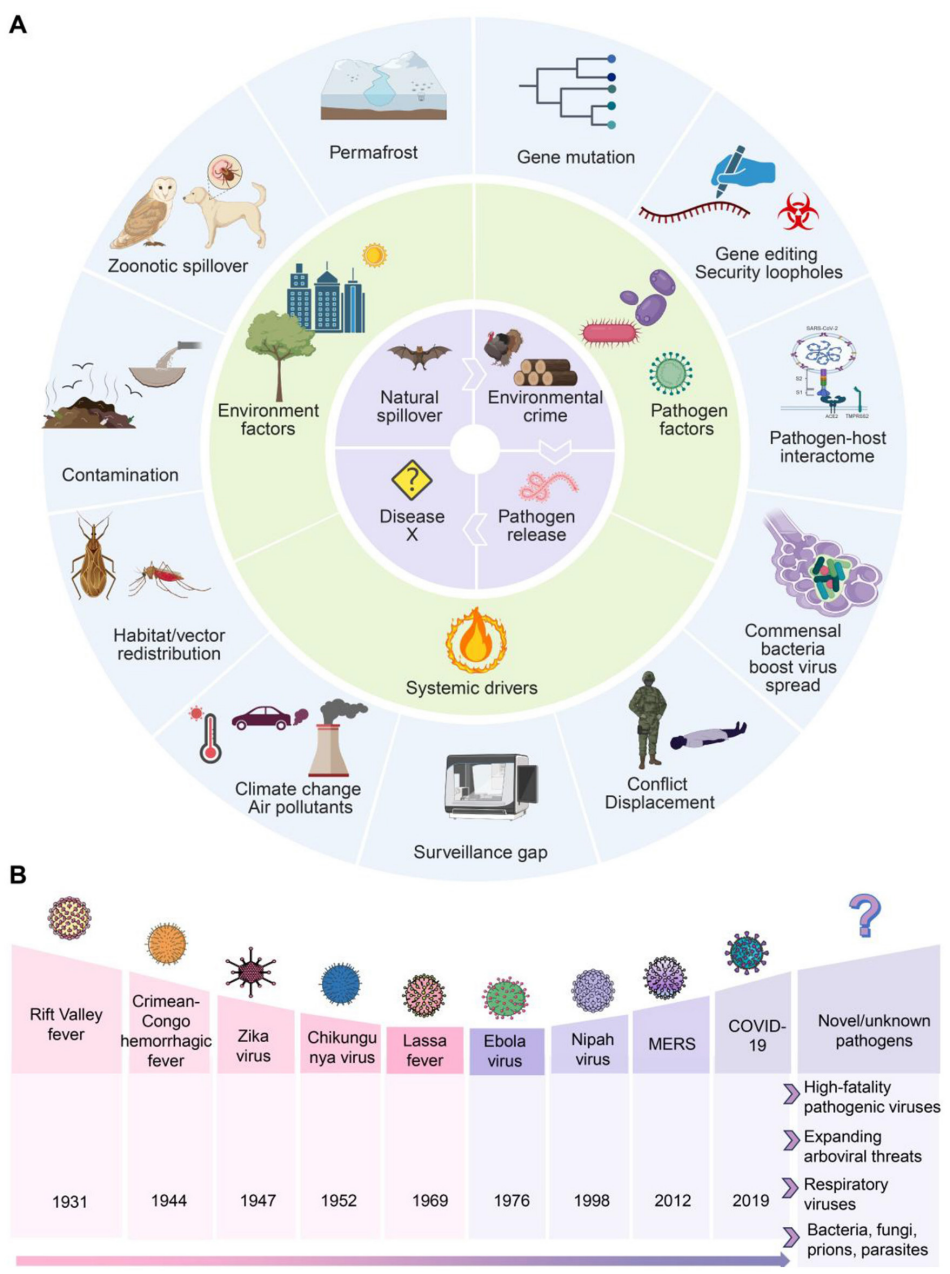
Origins, drivers, and potential pathogens of Disease X. **(A)** Potential origins and accelerating drivers of emergence. The threat of Disease X stems from a complex risk network comprising natural spillover, environmental crime, engineered pathogens, and systemic pressures. Natural spillover occurs primarily through wildlife trade and pathogen adaptive mutations; environmental crime creates high-risk contact interfaces within regulatory blind spots; and advances in biotechnology introduce the risk of accidental or deliberate pathogen release. These origins are amplified by systemic drivers such as climate change, ecological degradation, and surveillance gaps. Ultimately, they are accelerated by the virus's intrinsic evolutionary capacity—including key molecular mutations, the subversion of host mechanisms, and environmentally modulated stability—collectively driving the efficient spread of pandemics within a globalized context; **(B)** Spectrum of potential causative pathogens. Disease X encompasses a broad spectrum ranging from known high-risk pathogen families to entirely unknown or unidentified pathogens, necessitating a proactive, family-level perspective for research and preparedness. Key foci include respiratory viruses, highly pathogenic viruses, rapidly expanding arboviruses, bacteria, fungi, prions, and parasites. The most quintessential threat may ultimately arise from completely novel pathogens. **(A)** Image created using BioRender.

Ultimately, the most quintessential Disease X scenario may involve entirely novel or unidentified pathogens—from lower-risk families or unknown taxonomic groups. The WHO process explicitly allows for “Pathogen X” candidates within each viral family, recognizing that genetic shifts could abruptly elevate their threat. This reality is operationally reflected in outbreaks of severe acute illness of unknown origin, which expose critical gaps in surveillance and diagnostic readiness for truly novel agents (86). Together, the potential sources of Disease X span a hierarchical spectrum: from evolving members of known high-risk viral families and expanding arboviruses, to bacterial and fungal pathogens propelled by antimicrobial resistance, to prions with unique transmission and persistence characteristics, to parasitic agents with regionally significant epidemic potential, and ultimately to completely novel agents. Effective preparedness requires a nuanced, family-level understanding of the distinct virology, ecology, evolution, and resistance patterns characterizing these diverse groups. More information about the origins, drivers, and potential pathogens of Disease X pathogens are illustrated in Figure 1.

## 4 One Health surveillance and diagnosis feedback loop

A robust defense against Disease X relies on an integrated, closed-loop system built on a synergistic triad: proactive multi-layered surveillance, advanced agile diagnostics, and integrative operational frameworks that bind them into a dynamic detection-confirmation-response network.

The cornerstone is proactive, multi-layered surveillance, shifting from reactive to preemptive monitoring. This requires adopting One Health frameworks that systematically monitor the animal-human-environment interface to preempt zoonotic spillover events (87). A pivotal advancement is the move toward agnostic pathogen discovery through metagenomic sequencing of human, animal, and environmental samples, which can identify novel genetic material before an outbreak becomes clinically apparent (88). This is powerfully complemented by systematic immunomic profiling, where the adaptive immune system serves as a dynamic biosensor, recording antigen exposures through B cell and T cell receptor repertoires, revealing past and present pathogen encounters and aberrant immune states (89, 90). For specific threats, targeted approaches remain crucial: analyzing airborne transmission dynamics offers alerts for respiratory pathogens (91), while innovative platforms like 3D-printed microfluidic devices enable rapid detection of vector-borne pathogens (92). The integration of these diverse data streams—from wastewater-based epidemiology and genomic surveillance to immunomic landscapes—creates a multi-dimensional surveillance picture, shifting the paradigm from merely detecting pathogens to understanding population-level exposures and vulnerabilities (93–97).

Once a signal is detected, advanced and agile diagnostic technologies are critical for rapid confirmation and characterization. Conventional molecular diagnostics like RT-PCR remain essential for direct pathogen identification (98). For novel or complex scenarios, a multi-technology approach is key. Machine learning frameworks applied to multi-omics data, including immune receptor sequences, can screen for multiple diseases or pinpoint specific conditions from a single sample, aiding in differential diagnosis (90, 99–101). Sensitivity and multiplexing capability are paramount for early, low-burden infection. Digital detection technologies, such as Single Molecule Arrays (Simoa) and digital PCR, achieve exceptional sensitivity by confining and counting individual molecules, enabling measurement of low-abundance biomarkers (102–104). For multiplexing, technologies employing barcoded signal amplification or nanopore-based detection allow simultaneous quantification of multiple analytes (105, 106). Serological profiling through bead-based multiplex assays provides a broad view of antibody responses (107). The diagnostic arsenal is further strengthened by tools offering high specificity, such as CRISPR-based systems for nucleic acid detection, and by platforms that enable simplification and integration. Microfluidics and automation streamline workflows, reducing time and sample consumption (103, 108), while innovations like quantum dot-based lateral flow assays and integrated self-testing systems combine high sensitivity with point-of-care applicability (109–111). Together, these technologies—spanning ultra-sensitive molecular detection, specific probe systems (e.g., aptamers, engineered antibodies), and simplified, integrated platforms—open new dimensions for diagnosis through both direct pathogen detection and systematic host response profiling *via* liquid biopsy approaches.

Integrative Operational Frameworks are crucial to unify these surveillance and diagnostic strands into an effective response. The 7-1-7 global target provides an actionable timeline, where rapid diagnostic tests are pivotal for early containment. Achieving this target demands building regional diagnostic hubs with capacity for both high-throughput multi-omics analyses and rapid POCT deployment (112). Ideally, surveillance systems should correlate data from wastewater, clinical reports, genomic, and immunomic surveys; population-level multi-source data anomalies could trigger targeted sequencing for pathogen discovery and the deployment of multiplexed POCTs for confirmation, creating a dynamic feedback loop where surveillance guides diagnostic application and diagnostic findings refine surveillance targeting (113). However, operationalizing this loop presents challenges, including simplifying workflows for advanced assays, robustly validating AI models across diverse populations, and ensuring affordability (114). More critically, the effectiveness of such a closed-loop design is threatened by the risk of model-driven misjudgment when initial signals are embedded in high epistemic uncertainty. The gap between early diagnostic hypotheses and final diagnoses remains a persistent global health challenge. For instance, during the 2025 DRC outbreak, unexplained acute febrile illnesses initially attributed to viral hemorrhagic fevers were later reclassified after primary pathogens were excluded, illustrating how systemic biases, methodological limitations, and sociocultural factors can delay the identification of novel or unexpected threats (112). If decision-support algorithms process such ambiguous data deterministically, they risk triggering false-positive cascades or, conversely, overfitting to noise and overlooking genuine threats.

To address these uncertainties, a beneficial approach involves shifting the decision-making process from binary threshold-triggering to probabilistic reasoning. First, integrating explicit uncertainty quantification into predictive models—clearly distinguishing aleatory uncertainty from structural uncertainty—can enable the output of calibrated confidence intervals rather than definitive alerts (115). Second, operating the feedback loop on an adaptive Bayesian model averaging presents an effective strategy; here, initial surveillance anomalies can establish prior probabilities across competing predictive models, whose weights are then iteratively updated with subsequent diagnostic data to yield a weighted-averaged threat estimate. By explicitly accounting for model uncertainty and systematically considering multiple working hypotheses, this approach helps avoid premature escalation or dismissal driven by a single data point or premature cognitive anchoring (116). Third, for low-probability but high-consequence events, incorporating human-in-the-loop safeguards can serve as a critical mechanism for contextual adjudication and cognitive debiasing. While automation accelerates data fusion, having domain experts assess signal plausibility and strengthening communication networks between clinicians and public health personnel can be instrumental in contextualizing these signals before triggering resource-intensive containment measures (117). Finally, adopting a stance of robust adaptive management in decision policies—employing graduated, reversible interventions rather than irreversible, stringent measures—can help optimize trade-offs between the precautionary principle and the societal costs of false alarms. Developing sustainable local diagnostic capabilities and robust One Health surveillance platforms provides the foundation for executing such adaptive strategies. Importantly, drawing on past pandemics and historical misdiagnosis cases can effectively inform the development of this next-generation toolkit, highlighting the value of refining and tailoring diagnostic tools to recognize emerging pathogens within their complex ecological and social contexts (118).

**Figure 2:**
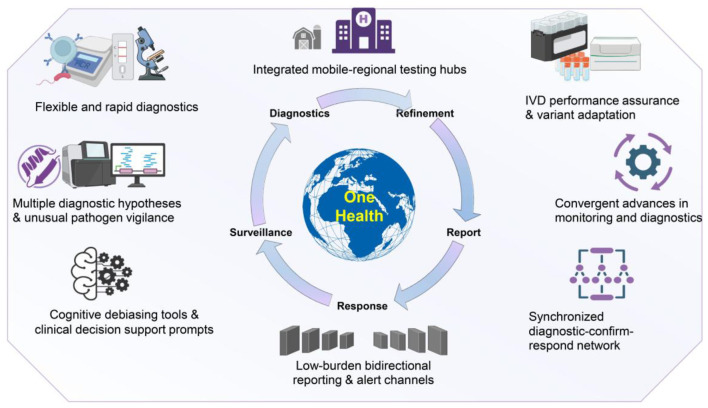
One Health surveillance-diagnostic feedback loop. This framework advocates for a multi-disciplinary, adaptive approach to pandemic preparedness that embraces probabilistic reasoning under uncertainty, moving beyond over-reliance on single disciplines or technologies. Its foundation lies in the integration of One Health principles, which systematically bridges human, animal, and environmental health sectors to enable holistic threat detection. This is achieved through technological synergy, combining rapid diagnostics, molecular sequencing (e.g., metagenomics), digital detection platforms, and AI-enabled analytics to enhance the sensitivity, speed, and scope of surveillance. Operationally, the system is designed for resilience, incorporating bidirectional reporting channels, cognitive-debiasing tools, and synchronized response networks to ensure timely and coordinated actions. These components function within an adaptive, closed-loop cycle wherein surveillance continuously informs diagnostic targeting, and diagnostic results in turn refine surveillance strategies—striking a dynamic balance between broad monitoring and focused investigation. Image created using BioRender.

To summarize, a robust defense against Disease X is built on an integrated, closed-loop system that synergizes advances in multi-source surveillance and multi-technology diagnostics. Proactive One Health monitoring, agnostic metagenomic sequencing, and population-level biomarker profiling (including immunomics) form a rich surveillance layer. This triggers a diagnostic phase employing a spectrum of tools—from CRISPR-based specific detection and ultra-sensitive digital PCR assays to microfluidics-enabled integrated platforms and multi-omics host response analysis—for precise, multiplexed, and accessible confirmation. The convergence of these elements within a synchronized operational framework—underpinned by probabilistic reasoning and human-in-the-loop oversight—creates a dynamic network wherein broad surveillance triggers targeted diagnostic inquiry, and detailed diagnostic results, in turn, refine and focus surveillance efforts. This mutually reinforcing cycle forms an essential, adaptable framework for mitigating the threat of an unknown, fast-moving pathogen. See more details in Figure 2.

## 5 Medical countermeasures

The threat of Disease X demands more than any single medical solution. As previously discussed, surveillance and diagnostics form the critical first layer of defense. Building on that foundation, an effective response relies on the seamless integration of vaccines and therapeutics. Rapid vaccine deployment is essential to establish broad immunity and curb transmission, while innovative therapeutics are key to reducing severe illness and mortality. Ultimately, it is the coordinated agility between these pillars—from detection to prevention and care—that determines the success of our response. Strengthening these interoperable capabilities remains an important strategy for mitigating the impact of Disease X.

### 5.1 Pivot role of vaccine platforms in Disease X preparedness

The development of vaccines against Disease X necessitates platforms that are both rapidly adaptable and capable of inducing broad, durable protection. Foundational technologies such as mRNA, viral vector, and subunit vaccines form the bedrock of rapid-response capabilities. The mRNA platform, exemplified by vaccines like ARCoV, which encodes the receptor-binding domain and demonstrates stability at room temperature for at least 1 week, offers unparalleled versatility and speed for initial deployment (119, 120). Innovations such as the MS2 bacteriophage capsid system, which enables one-step mRNA synthesis and packaging in Escherichia coli (E. coli), could significantly streamline production (121). Viral vector vaccines, leveraging diverse backbones such as adenovirus and poxvirus, support robust humoral and cellular immunity and are amenable to mucosal delivery, making them prominent candidates for emergency response (122). For broader global access and equity, traditional platforms like whole inactivated virus and protein-based vaccines remain critically important, especially for low- and middle-income countries, due to their strong safety profiles, proven efficacy, and established manufacturing pathways (123).

Beyond platform selection, the strategic design of antigens and immunization regimens is paramount. A critical frontier is the development of broad-spectrum or “universal” vaccines that confer protection across related viral genera or families (124, 125). Computational immunology and structural biology are key enablers here. For instance, reverse vaccinology and immunoinformatics approaches, which computationally design multivalent, multi-epitope vaccines by selecting key antigenic proteins and predicting immunodominant T- and B-cell epitopes, offer a rational and rapid design pathway for novel pathogens, as demonstrated in candidate designs for viruses like HSV-2. This *in silico* methodology, coupled with high-throughput screening of antigen variants, can accelerate the identification of conserved, immunogenic targets in the absence of prior structural information (126). Experimentally, sequential immunization strategies using virus-like particles derived from different SARS-CoV-2 variants have been shown to elicit superior neutralizing antibody and T-cell responses in mice, providing a prototype model for boosting breadth against emerging threats (127). Likewise, sustained co-delivery of antigens and adjuvants (e.g., *via* polymer-nanoparticle hydrogels) can enhance the magnitude, durability, and breadth of antibody responses against drifted strains (128).

Novel delivery and formulation technologies are equally vital for enhancing vaccine utility. Thermostable formulations, such as lyophilized protein vaccines for filoviruses and orally administered, VSP-decorated eVLPs for SARS-CoV-2, demonstrate complete protection in animal models while overcoming cold-chain limitations—a decisive advantage for equitable global distribution (129, 130). Intranasal delivery of subunit vaccines can induce protective mucosal IgA responses, potentially blocking infection at the portal of entry (131). Furthermore, platform refinements like photoactivated nanovaccines offer spatiotemporal control of immune activation, representing a groundbreaking approach with potential for precise immunotherapy (132).

The clinical evaluation and deployment of these vaccines require equally innovative frameworks. Heterologous prime-boost regimens (e.g., adenoviral vector prime followed by mRNA boost) have been shown to induce significantly higher antibody titers than homologous regimens, providing crucial flexibility for rapid global rollout amidst supply constraints (133). Clinically, the deployment of adaptive platform trials (APTs) with “ever-warm” designs and pre-established template protocols for Disease X is essential for accelerating efficacy evaluations during a crisis. These trials, exemplified by REMAP-CAP and RECOVERY, allow for the continuous, efficient assessment of multiple interventions (134, 135). Furthermore, collaborative online platforms that enable real-time living systematic reviews of vaccine safety and efficacy across special populations (e.g., pregnant women) are vital tools for rapid evidence synthesis during an emerging outbreak (136).

However, the promise of these advanced platforms should be balanced with a clear-eyed assessment of challenges. While mRNA-LNP technology is transformative, strategies to mitigate its potential toxicity require ongoing attention (137). The quest for broad-spectrum vaccines faces the fundamental biological challenge of viral antigenic diversity and immune escape (138, 139). Moreover, the ultimate success of any vaccine depends on sophisticated immune monitoring. Advances in single-cell technologies, such as single-cell RNA sequencing and fluorescence-activated cell sorting, are revolutionizing our understanding of protective immune correlates, like the role of tissue-resident memory CD8^+^ T cells in rapid recall responses against respiratory pathogens (140). Similarly, high-throughput profiling of B-cell repertoires, aided by machine learning models, can identify signatures of broadly neutralizing antibodies and predict antibody specificity, informing vaccine design (141, 142). Rapid, multiplexed point-of-care serological assays, including lateral flow immunoassays and biosensor microarrays, are also evolving to provide real-time, personalized assessments of humoral immunity, potentially guiding vaccination schedules (143, 144). These tools collectively move the field toward a more nuanced, data-driven understanding of vaccine-induced protection.

### 5.2 Therapeutic interventions

The therapeutic paradigm for Disease X necessitates a multi-pronged approach, combining broad-spectrum antivirals, biologics, and optimized supportive care within resilient health systems (145, 146). Within the One Health framework, a helpful approach to conceptualizing therapeutic interventions is to position them not merely as clinical countermeasures but as integral components of a holistic strategy bridging human, animal, and environmental health. Adopting this lens allows effective therapeutics to be utilized as targeted tools at multiple nodes of the spillover pathway—ranging from preventing amplification in animal reservoirs, to severing human-to-human transmission chains, and mitigating environmental contamination.

A fundamental strategic pillar is the pre-positioning of pathogen-agnostic platforms. Repurposing efforts include host-directed agents like nitazoxanide, which targets shared host pathways to stimulate interferon responses(145, 147). By targeting conserved host mechanisms rather than pathogen-specific structures, they may retain efficacy across diverse zoonotic pathogens and reduce the selective pressure that drives antimicrobial resistance in both human and animal populations (148). Beyond repurposing, innovative discovery methodologies, such as Bayesian optimization guided by Gaussian Process Regression, are being employed to efficiently identify combinatorial drug formulations with high virucidal efficacy (149). A comprehensive medicinal chemistry strategy advocates for combination therapies and multi-target agents to balance broad activity with specificity and overcome resistance (150).

Immunotherapies represent a rapidly advancing frontier, moving beyond traditional antimicrobials. Monoclonal antibodies have proven highly effective, with pan-ebolavirus immunotherapeutics demonstrating the potential for rapid post-exposure disease reversion (151). For Disease X, the identification of antibody classes with broad neutralization potential, such as those targeting conserved stem helices in betacoronaviruses and encoded by specific gene signatures (e.g., IGHV1-46/IGKV3-20), provides a blueprint for developing pre-emptive biologics (142). Furthermore, leveraging pre-existing cross-reactive memory B cells and naive B cells through vaccination can be a strategic avenue to accelerate antibody responses against novel pathogens (152). Nevertheless, candidate biologics should be evaluated not only for human efficacy but also for their potential utility in animal reservoirs to intercept zoonotic spillover at its origin (153).

Therapeutic strategies are increasingly harnessing fundamental immune mechanisms. Modulation of innate-like T cells, such as invariant Natural Killer T (iNKT) cells *via* the CD1d axis, is being explored as an adjunctive therapy to potentiate immune responses against intracellular pathogens (154). Similarly, novel delivery systems like miR-107-enriched exosomes have shown promise in modulating host cell pathways (e.g., ROS/Wnt/autophagy) to inhibit intracellular bacterial growth and attenuate infection (155). Another sophisticated approach involves dendritic cell (DC)-targeting vaccines, where antigens are fused to antibodies against specific DC surface receptors. This strategy, enhanced by *in silico* epitope mapping to select the most immunogenic and conserved targets, aims to directly program antigen-presenting cells to elicit superior T- and B-cell responses, offering a versatile platform for rapid vaccine development (156). Offering cross-species mechanisms, these interventions represent a critical advance in pandemic preparedness by enabling strategies that address the full ecological scope of disease emergence (157).

Integrating systems immunology into therapeutic strategies is critical for patient stratification and mechanistic insight. Machine learning-enhanced immune receptor sequencing identifies pathogen-specific T-cell biomarkers, assessing population-level immune exposure and memory (158). When combined with single-cell profiling, this offers a multidimensional view of host-pathogen interactions to guide personalized interventions (159). Extending these tools to animal populations, alongside environmental pathogen surveillance, establishes a cross-sectoral feedback loop. Together, these approaches bridge clinical medicine, veterinary science, and ecosystem monitoring, enabling dynamic assessment of therapeutic efficacy and timely protocol refinement across the human-animal-environment continuum (157).

### 5.3 Challenges and integrated path forward

A cohesive defense against Disease X requires the strategic synergy and sequencing of vaccines and therapies, underpinned by robust immunological insight. Mathematical modeling indicates that broadly protective vaccines, even if not fully sterilizing, can play a crucial role by reducing the effective reproduction number, slowing epidemic growth, and buying time for health system mobilization and the development of pathogen-specific countermeasures. For example, modeling of a broadly protective sarbecovirus vaccine suggests that pre-emptive vaccination of high-risk groups could significantly reduce mortality during the initial phase of a novel SARS-like outbreak (160). This modeling underscores the value of pre-positioning prototype vaccine libraries against virus families with shared structural properties (124).

The path forward is inherently interdisciplinary. It requires the integration of basic discovery—such as the rapid response subunit vaccine design pipelines that enabled swift COVID-19 candidate development—with advanced clinical trial frameworks like APTs (135). Regulatory science should evolve in parallel, streamlining processes through pre-established protocols while addressing emerging safety considerations for novel platforms (137). Furthermore, enhancing supply chain resilience for both vaccines and therapeutics necessitates integrating AI-driven risk forecasting with robust logistics planning (161). Crucially, global collaboration and equitable access frameworks should be cemented in peacetime to ensure these technologies fulfill their lifesaving potential during a crisis (162).

**Figure 3:**
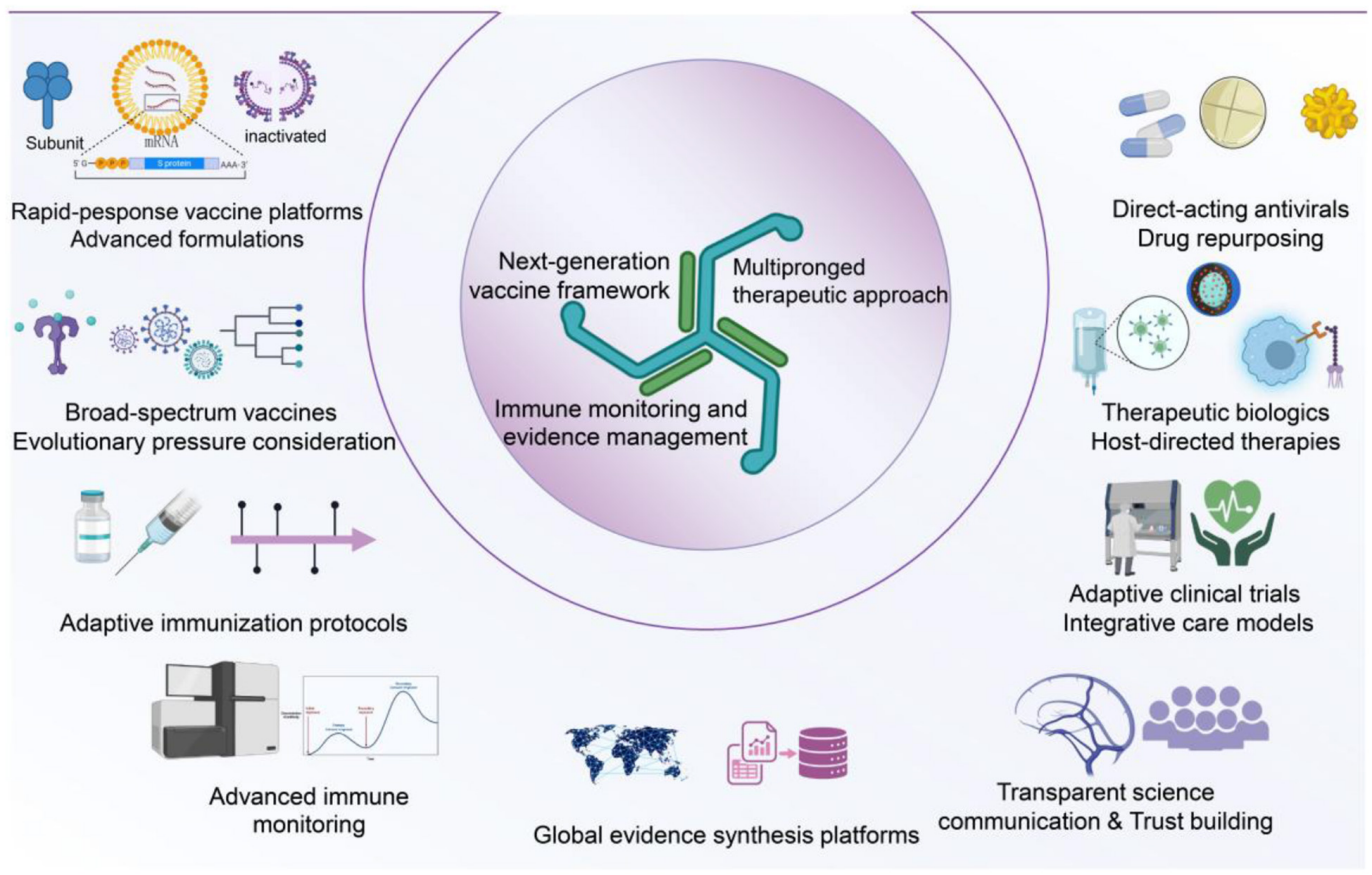
Medical countermeasure system. Structured around vaccines and therapeutics, this system can be integrated through a One Health lens to address cross-species threats. Vaccine development leverages rapid-response platforms—such as mRNA and viral vectors—enhanced by broad-spectrum designs and thermostable formulations applicable to both human and animal reservoirs. The therapeutic arm expands beyond conventional antivirals to employ host-directed strategies targeting conserved immune pathways across species, including immune modulation, nanoparticle-facilitated delivery, and *in silico*-optimized designs. These components are underpinned by enabling infrastructure, such as adaptive clinical trials and cross-sectoral evidence synthesis, ensuring agile and resilient health defense capabilities. Image created using BioRender.

Looking ahead, critical gaps remain in pandemic preparedness. These include a lack of validated preclinical correlates of protection against unknown pathogen antigens and the absence of a robust, globally deployed real-time immunogenicity monitoring system for post-deployment surveillance. Promisingly, emerging fields like microbiome therapeutics highlight a broader view of host defense, suggesting interventions that modulate commensal microbes to promote physiological resilience and complement traditional antimicrobial strategies (163). Addressing these gaps through proactive framework development, sustained basic and translational research, and an unwavering commitment to equity will ultimately determine the world's capacity to mount an effective, immunologically informed response to the next Disease X. Further strategies are presented in Figure 3.

## 6 Non-pharmaceutical interventions and prevention strategies

Non-pharmaceutical interventions (NPIs) form a critical, yet inherently limited, first line of defense against pandemic threats. Their optimal efficacy is realized not in isolation but when embedded within a coherent, multi-layered framework. This framework integrates immediate containment actions with proactive ecological prevention and is ultimately sustained by a deep understanding of socio-behavioral determinants.

The foundational layer of this framework focuses on coordinated containment and adaptive surveillance. Initial measures, such as risk-adjusted border controls informed by digital tools, aim to delay transmission (164). These are underpinned by surveillance systems that enable systematic threat prioritization and global oversight, as exemplified by national risk assessments and proposals for coordinated laboratory networks (165). The calibration of such travel-based policies can be guided by modeling that quantifies their impact on domestic outbreak dynamics and the substantial healthcare resources required to sustain them (166).

To transition from reactive containment to sustained resilience, the framework should incorporate a second layer dedicated to proactive ecological governance and spillover prevention. This is anchored in robust One Health coordination, establishing interconnected surveillance networks with standardized protocols and advanced tools for pathogen monitoring (167). Ecological management—encompassing wildlife trade regulation, sustainable land use, and genomic surveillance of shifting pathogen landscapes—seeks to reduce high-risk human-animal interfaces (42). For vector-borne threats, novel biocontrol strategies such as Wolbachia-based interventions offer a sustainable approach, while more advanced gene-drive systems remain under careful evaluation, necessitating thorough risk-benefit analysis (168–170). Environmental monitoring remains crucial, as studies reveal persistent pathogen evolution in reservoirs like wastewater and highlight transmission risks in agricultural settings, demanding integrated environmental controls (171).

The third and enabling layer addresses the systemic, technological, and human factors that determine the feasibility and longevity of NPIs. Operational readiness is supported by innovations like smart mobile hospitals for rapid response (172) and models that guide the tiered deployment of community interventions based on epidemiological parameters (173). Technological applications, such as smart masks for health monitoring, illustrate the evolution of protective equipment (174). However, long-term success hinges on socio-behavioral integration. Research indicates that adherence is best predicted by an interplay of motivation, capability, and supportive environmental factors like clear guidance and institutional support, cautioning against narratives of pure personal responsibility (175). Public engagement reveals a demand for protective early action, balanced guidance, and governmental agility (176). Furthermore, the disparate mental health impacts on vulnerable groups and the significant economic trade-offs of measures like school closures underscore the necessity of equity-sensitive policy design (177, 178). Systemic resilience is further strengthened through cross-jurisdictional partnerships that build coordinated response capacity (179).

**Figure 4:**
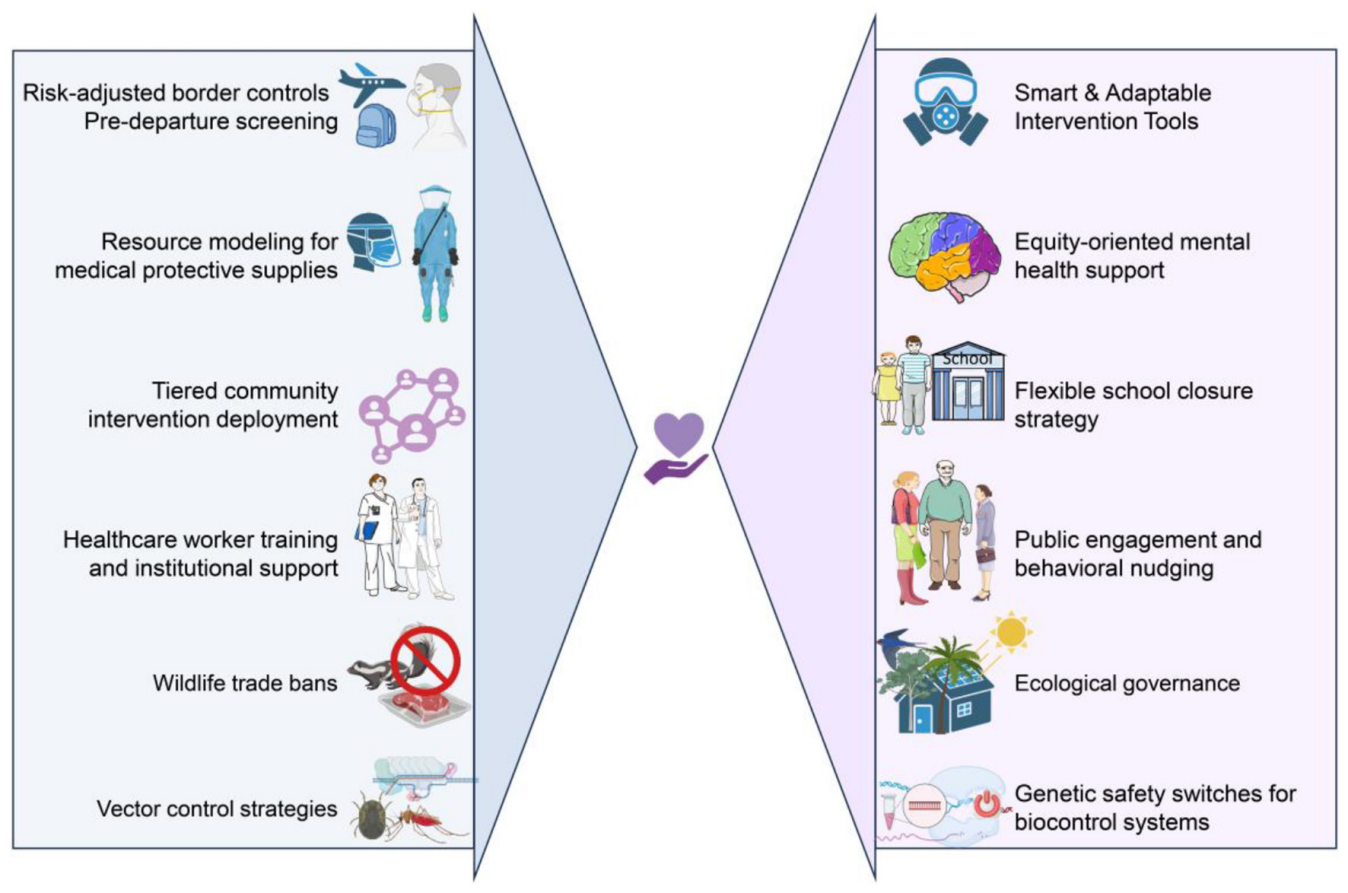
Sustainable non-pharmaceutical intervention and prevention framework. This framework requires the integration of containment, ecological management, and societal enablement to enhance systemic resilience. Coordinated containment and surveillance need to employ risk-adjusted border controls, pre-departure screening, and resource modeling to limit cross-border spread and ensure supply readiness. Dynamic outbreak tracking and delayed transmission further depend on tiered community interventions, vector control, and digital monitoring. Ecological management, guided by a One Health approach, should reduce spillover risks through wildlife trade restrictions and targeted environmental governance. Feasibility and sustainability should be advanced by incorporating technological innovations—such as genetic safety switches—alongside socio-behavioral measures, including healthcare worker training, adaptable tools, equity-focused mental health support, public engagement, and flexible school policies. Together, these components form a coherent defense pillar that aligns immediate response with ecological prevention and societal empowerment, while consistently addressing equity and well being. Image created using BioRender.

In conclusion, an effective NPI strategy for Disease X should be conceptualized as an integrated framework. This framework spans coordinated containment and adaptive surveillance, proactive ecological and vector management, and operational and socio-behavioral enablement. Ultimately, it aims to transform NPIs from a collection of discrete measures into a coherent, scientifically informed, and socially sustainable pillar of pandemic defense. More details are depicted in Figure 4.

## 7 Infrastructure strengthening

An effective infrastructure framework for pandemic response should be conceived as an integrated system, where effectiveness is determined by the synergistic function of its foundational dimensions under acute stress rather than by their isolated performance. This necessitates a design that prioritizes adaptability, equity, and foresight across all interconnected components.

Supply chain resilience forms a critical backbone, ensuring the secure flow of essential materials. Its strength relies not merely on inventory but on institutionalized processes for systemic risk management. Longitudinal research, such as the study of the Colombian Air Force supply chain, demonstrates that proactive vulnerability identification directly enhances operational adaptability. A key insight is that acute stress, as experienced during a pandemic, can itself sharpen organizational awareness of weaknesses, catalyzing a diagnostic process that enables more targeted fortification of critical supply lines (180). Proactively, data analytics and predictive modeling offer tools to anticipate and mitigate disruptions in systems like pharmaceutical supply, though their utility may require adaptation during periods of deep uncertainty (181). This dimension is inextricably linked to governance, as securing supply chains is fundamental to operationalizing equitable resource distribution.

Operational health systems constitute the tangible delivery mechanism, where resilience is engineered through scalable clinical pathways and deliberate physical redundancy. Evaluations demonstrate that targeted investments in scalable service pathways, such as adaptable ambulance services, can preserve access to critical care during surges (182). Concurrently, analyses of core physical infrastructure, exemplified by a study of intensive care unit systems in Brazil, substantiate the strategic value of designing “slack” or built-in redundancy into foundational systems like oxygen supply, electrical capacity, and spatial configuration. This yields actionable principles for aligning surge capacity with clinical needs and for updating regulations to incentivize such resilience investments (183). However, this operational capacity can be undermined if broader preparedness plans lack detailed implementation strategies for critical enablers like financing and workforce, a gap noted in analyses of national influenza plans (184). Furthermore, resilience depends on sustainable medical logistics; the strain on personal protective equipment supplies during COVID-19 highlighted the need for circular-economy approaches to device and waste management to mitigate future environmental and supply pressures (185).

Underpinning both supply chains and clinical operations is evidence infrastructure, which provides the agile knowledge ecosystem required for rapid, informed decision-making. Initiatives like the German COVID-19 evidence ecosystem (CEOsys) provide a proof-of-concept, showing how pre-established, coordinated research networks can rapidly generate and translate living evidence to guide policy (186). The broader imperative is to build and routinely stress-test such integrated infrastructures—with dedicated functions for surveillance, synthesis, and communication—during inter-pandemic periods. This ensures they can effectively bridge the research-policy gap when emergencies strike, a core tenet of initiatives like Germany's PREPARED (187).

Ultimately, the functionality of these dimensions is governed by the principles of systemic risk governance, which should proactively address equity and adaptive management. A focus on formal equality in resource distribution, as seen in some aspects of the U.S. COVID-19 response, can exacerbate disparities by overlooking structural barriers. Equitable design, therefore, requires using data-driven tools to overcome such outcomes (181). This governance layer is responsible for integrating insights from the evidence infrastructure, managing trade-offs identified by supply chain risk processes, and ensuring that operational health system designs are inclusive and responsive to community needs.

**Figure 5:**
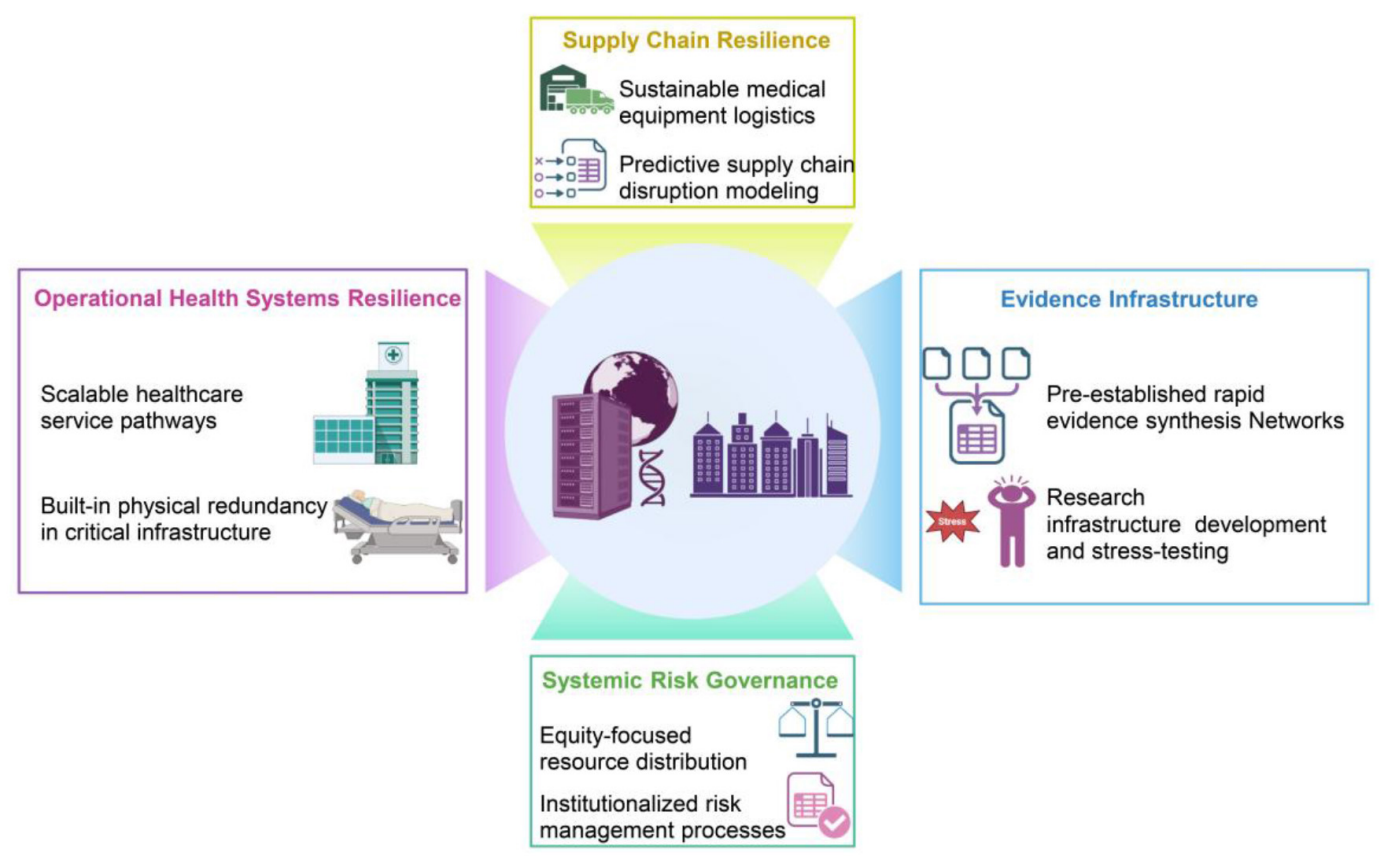
Critical infrastructure strengthening requirements. The overall resilience of the framework requires synergistic integration across four major dimensions. Supply chain resilience needs to be enhanced through sustainable medical logistics and predictive disruption modeling to ensure the continuous supply of essential resources. The operational resilience of health systems ought to be strengthened by developing scalable service pathways and incorporating physical redundancy into critical infrastructure for adaptive response. Evidence infrastructure should be reinforced *via* pre-established rapid evidence synthesis networks and stress-tested research systems to enable robust, data-driven decision support. Furthermore, systemic risk governance requires advancement through equity-focused resource allocation and formalized risk management processes to promote proactive and equitable risk mitigation. Image created using BioRender.

In essence, true preparedness emerges from the seamless interconnection of these dimensions: resilient supply chains secured through institutionalized risk management; operational health systems engineered for surge capacity and sustainability; a pre-emptive knowledge ecosystem for rapid evidence synthesis; and an overarching governance framework that ensures adaptive and equitable implementation across the entire system. The major requirements are further specified in Figure 5.

## 8 Modeling frameworks

Robust modeling frameworks are essential for guiding preparedness and response to Disease X, providing a structured approach from early warning to strategic policy evaluation. These models form an integrated hierarchy, yet their application involves inherent trade-offs and should be situated within the complex process of translating evidence into policy.

At the onset of Disease X, predictive surveillance models can provide vital early warnings, though forecasting emergence itself remains unfeasible due to socio-ecological complexity (188). Upon detection, transmission models—historically hobbled by parameter sensitivity and static assumptions in novel outbreaks—can benefit significantly from prompt adaptation (189). Because initial pathogen characteristics are largely unknown, relying on static parameterization often proves impractical; instead, forecasting frameworks can gain a significant advantage by balancing mechanistic integrity with statistical flexibility, reorienting the stochastic-deterministic trade-off toward rapid, adaptive iteration (190). The COVID-19 pandemic specifically highlighted the complexity of these unknowns, demonstrating that forecasting difficulties typically stem from an interplay of interrelated factors, including viral characteristics, evolving population immunity, and the dynamic intersection of public policy and human behavior.

As data streams in, Adaptive Bayesian learning and data assimilation offer valuable methods to transition models from priors to data-driven posteriors, dynamically updating critical unknowns like the effective reproduction number to correct early mis-specifications (191, 192). Concurrently, real-time Bayesian phylodynamics can be utilized to update transmission chains from genomic sequences without computational bottlenecks, while AI-driven metagenomics can extract functional traits (e.g., pathogenicity markers) to compensate for the absence of wet-lab data (193–195). To complement these quantitative approaches, large language model frameworks provide a powerful mechanism for rapid iteration through zero-shot text reasoning (196). When facing previously unseen pathogen features—such as the emergence of a novel variant whose epidemiological profile is not yet quantified—these models can incorporate real-time textual reports from authoritative sources regarding infectiousness, severity, and immune evasion. This capability facilitates immediate model adaptation, bypassing the delays associated with extensive data accumulation or full model retraining.

To address multi-wave realities, dynamically adjusting parameters such as the infection probability and mobility restrictions within the random-walk Monte Carlo framework is highly effective; rather than requiring fundamental architectural recalibration, modulating these parameters based on the timing and duration of interventions captures epidemiological regime shifts—such as surges driven by relaxed constraints or new variants. Furthermore, when fundamental shifts in disease dynamics occur (as evidenced by the Omicron variant's ability to escape vaccine-induced immunity), incorporating a small fraction of reinfections into the susceptible population and adapting infection probability to account for waning immunity play a crucial role in recapturing transmission trajectories and predicting subsequent waves (197).To support decision-making under such deep uncertainty, model confidence levels can serve as robust indicators of predictive reliability. By establishing confidence thresholds, policymakers can leverage a flexible metric to gauge the trustworthiness of forecasts, allowing them to strategically adopt or weigh predictions even when pathogen characteristics are only partially resolved (196). Ultimately, maintaining this iterative loop—augmented by multimodal intelligence that integrates policy context, mobility data, and real-time textual reasoning—helps ensure that decision-making evolves in lockstep with the unfolding reality of the pathogen (198).

For operational response, accurate forecasting of healthcare burden is critical. Hybrid approaches that combine techniques like Empirical Mode Decomposition with Long Short-Term Memory networks have demonstrated high accuracy in predicting hospitalizations, enabling proactive resource allocation (190). Concurrently, machine learning models can support clinical triage by rapidly identifying patients at higher risk of severe outcomes. Specialized models also address specific transmission pathways. For instance, computational fluid dynamics coupled with neural networks can synthesize complex physics into simplified predictive models for airborne transmission risk, informing protocols on distancing and ventilation, though their accuracy may be constrained by current limitations in foundational data and computational demands (199, 200).

At the strategic level, modeling supports policy evaluation and feasibility assessment. Intervention analysis of time series data can quantify the mortality impact of pandemics and policy responses (201). Mathematical modeling of lockdown exit strategies, incorporating variant dynamics and vaccination, highlights the importance of carefully timing the relaxation of NPIs to mitigate subsequent hospitalization waves (202). Furthermore, economic feasibility modeling indicates that meeting proposed investments in preparedness would require significant budget reallocations and donor aid, necessitating the prioritization of high-impact interventions under current financial constraints (203).

The integration of diverse predictive approaches can enhance preparedness. The convergent validity between model-derived zoonotic risk maps and expert qualitative assessments supports their combined use for targeting countermeasures (204). Ultimately, the translation of model outputs into policy is a complex socio-technical process. As analyses of responses to COVID-19 illustrate, models often function not as definitive facts but as fluid evidence within contested policy debates. The challenging effort to build a manageable “scientific consensus” from uncertain projections—exemplified by contested lockdown decisions—underscores the need to reconceptualize evidence-based policy beyond mere technical management (205).

**Figure 6:**
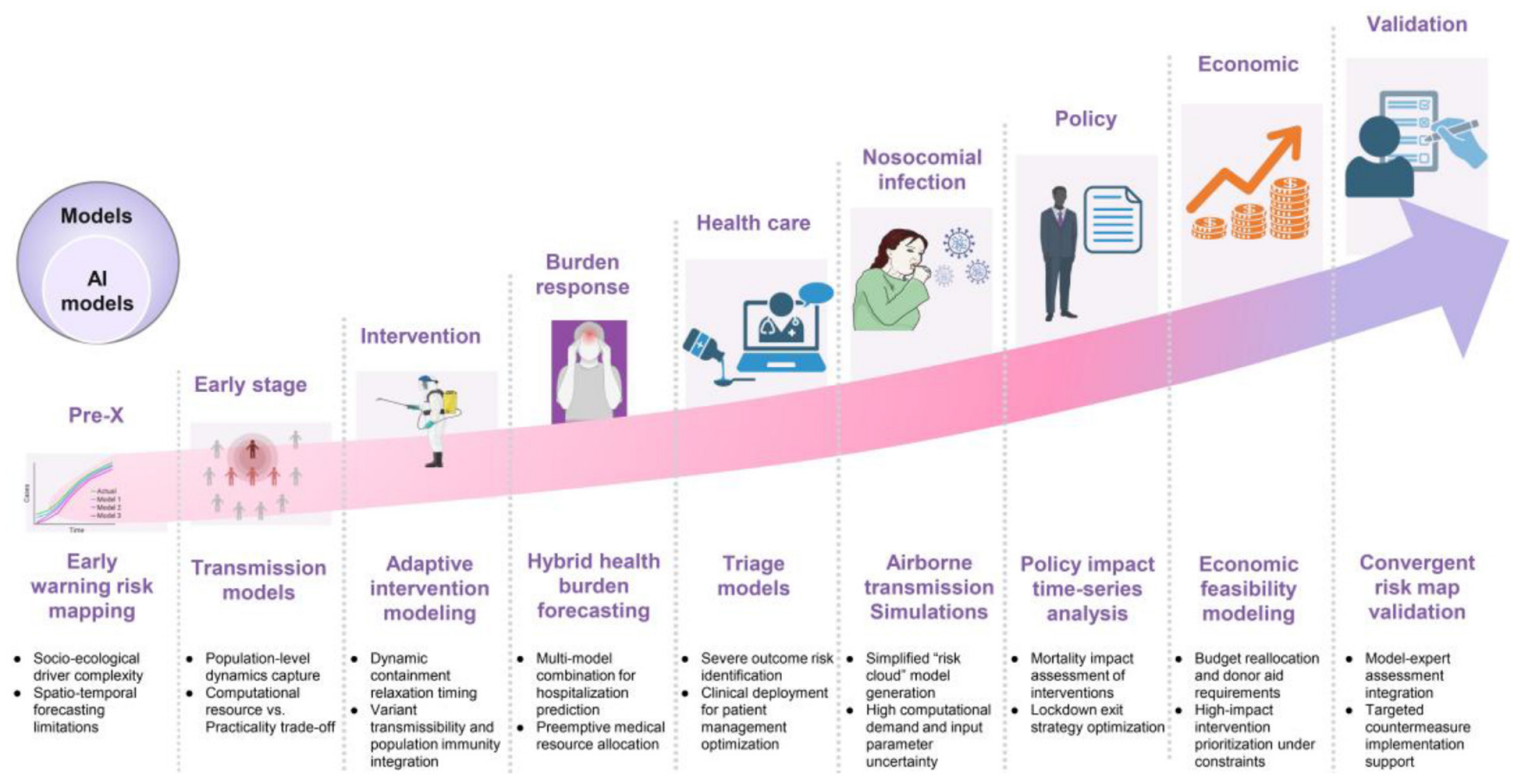
Predictive modeling approaches for Disease X. This framework establishes a hierarchical modeling system—from early warning to policy evaluation—for addressing Disease X. Surveillance models identify potential threats, while transmission dynamics models weigh intervention pathways. Operationally, hybrid forecasting and machine learning support resource allocation and environmental controls; strategically, models quantify policy impact and economic feasibility. When pathogen characteristics are initially unknown, the framework can enable rapid model iteration: adaptive Bayesian learning transitions models from prior assumptions to data-driven posteriors as information arrives; large language model frameworks allow zero-shot reasoning from real-time qualitative reports; and AI-driven phylodynamics can rapidly update transmission chains. For multi-wave dynamics, modulating parameters within Monte Carlo frameworks capture regime shifts without architectural recalibration. By integrating these diverse methods, treating outputs as fluid evidence, and applying adjustable confidence thresholds, decision-makers can navigate deep uncertainty to translate technical insights into adaptive policies. Image created using BioRender.

Collectively, an effective modeling framework for Disease X should integrate early-warning surveillance, dynamic transmission control, healthcare burden forecasting, and economic impact assessment. Its value lies not only in technical refinement but also in acknowledging the inherent uncertainties and trade-offs within models, and in navigating the critical process of translating their insights into coherent, adaptive policy. Key predictive modeling approaches for outbreak scenarios are presented in Figure 6.

## 9 Evidence challenges and data sharing

An effective pandemic response relies on a robust data ecosystem capable of generating, sharing, and translating evidence into timely policy. The experience of COVID-19, however, revealed that this ecosystem faces interconnected challenges in equity, integrity, and governance, which should be addressed through a coherent framework to prepare for Disease X.

The foundation of this ecosystem lies in adaptable surveillance networks and specialized databases. Systems like the WHO's GISRS demonstrate the value of established international platforms for pathogen tracking and repurposing (206). Advanced analytics applied to such data can yield critical public health insights (207), while curated domain-specific databases support foundational research, albeit with notable gaps such as the exclusion of viral pathogens (208). Yet, the utility of data depends not merely on its collection but on its equitable and effective sharing.

Systemic hurdles persistently undermine this process. The rapid proliferation of data platforms during the pandemic often resulted in siloed resources concentrated in high-income countries, with clinical data frequently lacking compliance with FAIR principles, demonstrating that volume alone does not ensure quality or equitable access (209). Ensuring data reliability amidst underreporting and political interference remains a critical challenge (210), while accelerated science exposed vulnerabilities in the evidence-to-policy pipeline, including the complex role of preprints and institutional data practices (211).

The integration of novel data streams, particularly genomic sequencing, intensifies these challenges and crystallizes critical issues of governance, quality, and equity. While essential for surveillance, genomic platforms have been scrutinized for governance opacity and accessibility barriers (212). Technical artifacts from decentralized global sequencing can distort phylogenetic analyses, necessitating improved standardization tools (213). Most critically, stark global disparities in sequencing capacity and data submission timeliness create an inequitable preparedness landscape and can delay risk assessment from animal reservoirs (214). These tensions manifest acutely during crises, as illustrated when rapid data sharing led to punitive travel bans, damaging international trust, and highlighting the need for governance that balances open science with protection from socioeconomic harm (215). Historical discrepancies in data release further underscore the credibility challenges within the sharing ecosystem (216).

Promising models point toward solutions. National initiatives demonstrate how FAIR-compliant platforms with integrated analytics and clear governance can support coordinated data sharing and real-time communication (217). Proactively addressing recognized data gaps, such as those for specific populations, is also essential (218).

**Figure 7:**
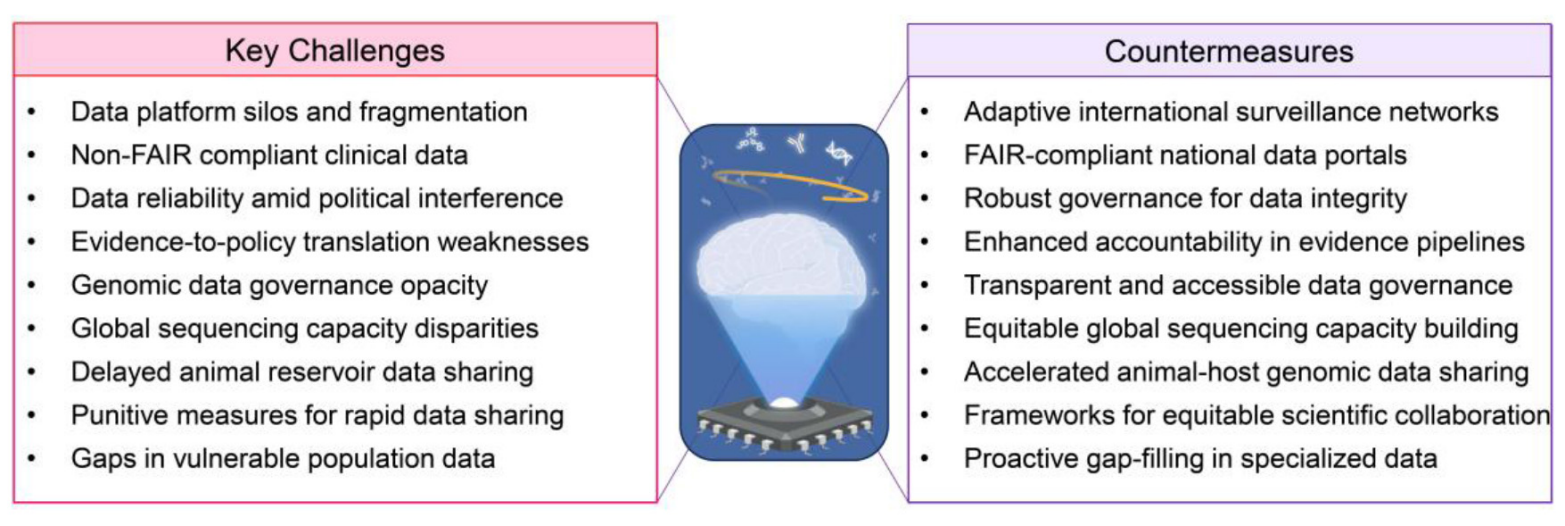
Data sharing challenges and countermeasures. Image created using BioRender.

To summarize, the outlined lessons define the essential requirements for a Disease X-ready data framework. This framework should extend beyond technical data collection to emphasize equitable access and capacity building, thereby bridging critical global disparities. It further requires strong governance and technical standards to ensure data integrity, transparency, and FAIR compliance, while carefully navigating the inherent tension between rapid data sharing and equitable collaboration. Critically, the framework should establish resilient translation pathways to effectively channel evidence into policy and action. Building such an integrated ecosystem is fundamental for enabling trustworthy global threat monitoring and response. A summary of data sharing challenges and corresponding countermeasures is provided in Figure 7.

## 10 Global collaboration and equity consideration

A resilient and equitable global response to Disease X requires a coherent governance framework that integrates cross-border coordination, equitable countermeasure access, and inclusive community engagement. Current efforts are often fragmented, with institutional and financial commitments remaining precarious (219). While multilateral negotiations for a pandemic agreement represent progress, proposed governance structures may face challenges in accountability and enforceability, potentially relying heavily on technical committees rather than strengthened political oversight (220). This governance complexity directly impacts border management. Although digital surveillance tools can enhance control, their effectiveness depends on rapid deployment and integrated data systems (165). Policy often over-emphasizes broad travel restrictions, whereas evidence suggests cross-border movements are more significantly influenced by the stringency of a destination's internal containment measures than by international travel bans (221). Border-centric approaches can carry severe human costs, exacerbating hardships for cross-border workers and inflicting dignity violations on vulnerable migrant populations, highlighting the need for context-specific strategies actively shaped by local communities (222).

Concurrently, accelerating countermeasure deployment necessitates streamlined regulatory pathways, though lower-income countries often face significant navigation challenges (223). Effective coordination depends on depoliticized scientific advice, WHO-led platforms, and prioritized resource allocation to strengthen basic health capacities in resource-poor nations (224). This underscores a central tension: effective control requires cross-border coordination, yet achieving this without exacerbating inequities demands a politically robust and equity-centered framework.

Indeed, pandemic resilience is fundamentally undermined without a steadfast commitment to equity. A focus on formal equality in resource distribution risks exacerbating existing disparities by ignoring structural barriers. An equity approach necessitates mechanisms to counter hoarding, such as regional stockpiles and equitable manufacturing hubs, alongside strengthening health systems through International Health Regulation reforms (42). The consequences of inequity are profound, with healthcare workers in under-resourced settings facing disproportionate risks (225), and mental health impacts disproportionately affecting women, youth, and low-income groups (226). Socially marginalized populations, including essential workers and homeless communities, have been consistently overlooked, experiencing amplified insecurity and access disparities (227).

Vaccine inequity, starkly illustrated by the COVID-19 pandemic, stems from a confluence of factors including vaccine nationalism, intellectual property constraints, limited manufacturing capacity, and fragile health systems (228). The global value of vaccination is immense, with conservative models attributing trillions in economic benefits from preserved GDP and averted costs (229). Financing, pipeline sustainment, and equitable access remain principal hurdles. Public-private partnerships have proven essential for rapid medical countermeasure development and should be enhanced for future preparedness (134). Sustained financing is critical not only for R&D but also for pre-pandemic manufacturing investments and equitable delivery infrastructure (160). Organizations like CEPI spearhead global coordination to establish rapid vaccine pipelines, including through pre-emptive platform validation (230), while Gavi supports equitable introduction and maintains emergency stockpiles (231, 232). The economic argument for equity is strong, as persistent inequity incurs high costs in preventable deaths, prolonged economic disruption, and increased variant risks, making cooperation a collective imperative (233).

**Figure 8:**
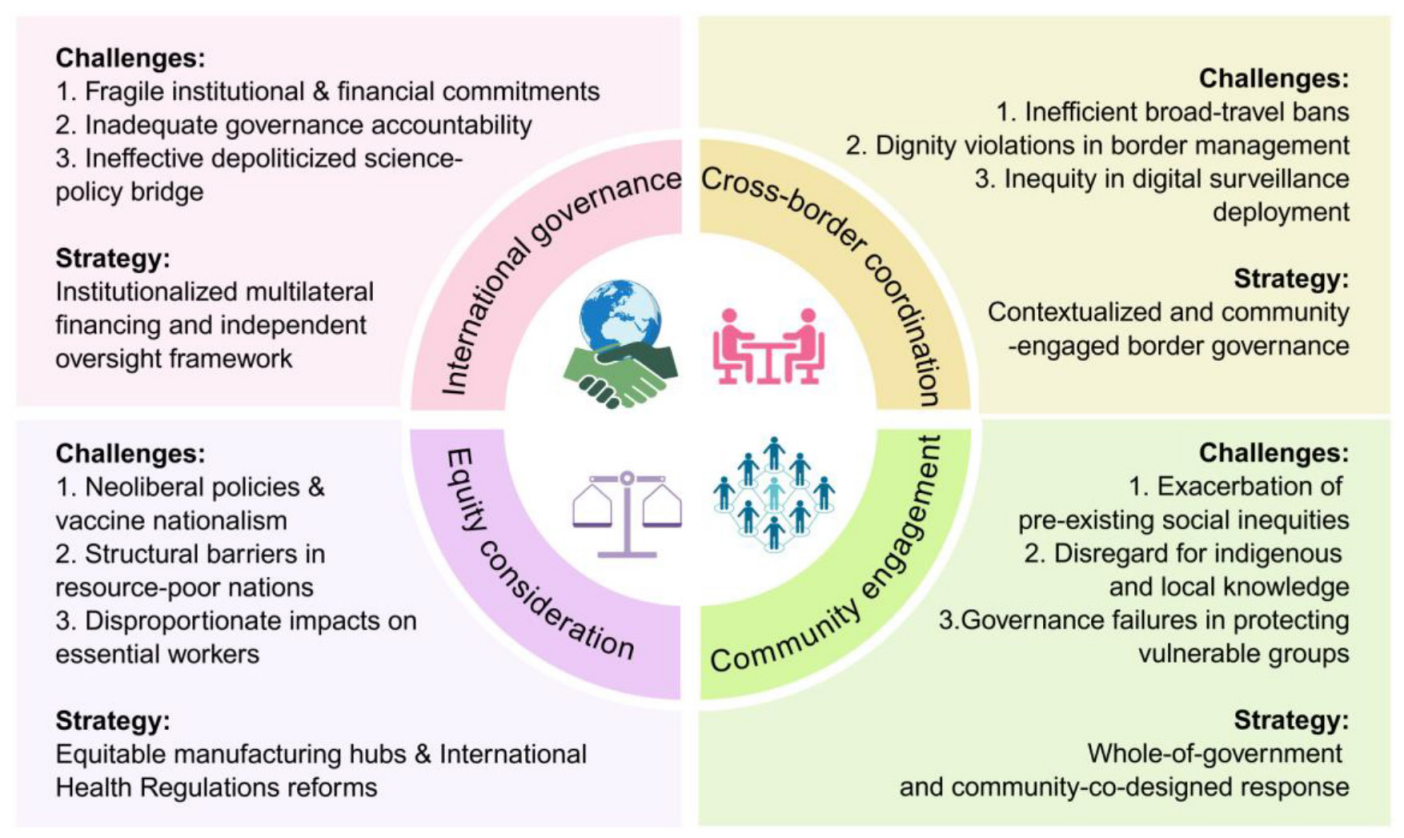
Global collaboration and equity considerations in Disease X preparedness. Image created using BioRender.

Therefore, an effective framework should be grounded in inclusive, culturally informed community engagement. Co-designing communication strategies with resource-poor communities empowers them to address local challenges (234). Indigenous communities' adaptive strategies, leveraging local knowledge, underscore the importance of supporting locally tailored resilience (235). Such community-academic partnerships are vital for building trust and addressing disparities, though they require sustained structural support (236). Ultimately, safeguarding vulnerable groups demands a “whole-of-government” approach that embeds equity in all policies, expands fiscal space for health, and employs tailored, multi-stakeholder strategies to bridge protection gaps (237). Figure 8 outlines the key challenges and strategic considerations for advancing global collaboration and equity.

## 11 Conclusion

Overall, the proposed framework—integrating agile technologies with a robust pandemic defense ecosystem—provides a comprehensive blueprint for countering Disease X. However, its viability under extreme conditions remains constrained by the risk of model-driven misjudgments amidst high epistemic uncertainty, systemic data integrity compromises under political or operational stress, and severe disruptions to global medical supply chains. To operationalize this framework at the system level, transitioning from theoretical integration to pragmatic resilience appears to be a crucial next step. This transition could be facilitated by establishing closed-loop surveillance-diagnostics feedback mechanisms that employ robust adaptive management—specifically, utilizing graduated, reversible interventions to balance the precautionary principle against the societal costs of false alarms. Additionally, it would be beneficial for systems to routinely stress-test infrastructural surge capacity during inter-pandemic periods and institutionalize circular-economy approaches for critical medical logistics to mitigate acute supply deficits. At the global level, practical actions would ideally shift equitable collaboration from an ethical aspiration to an operational standard. This could involve cementing pre-negotiated access frameworks and harmonized regulatory protocols during peacetime, adhering to strict FAIR-compliant data governance to bridge technological disparities between regions, and co-designing culturally informed communication strategies with resource-limited communities. Ultimately, translating these innovations into enforceable, cross-jurisdictional architectures is key to transforming the lessons of past crises into reliable safeguards against future unknown threats.
